# Scaffold dependent histone deacetylase (HDAC) inhibitor induced re-equilibration of the subcellular localization and post-translational modification state of class I HDACs

**DOI:** 10.1371/journal.pone.0186620

**Published:** 2017-10-18

**Authors:** Thomas W. Hanigan, Taha Y. Taha, Shaimaa M. Aboukhatwa, Jonna Frasor, Pavel A. Petukhov

**Affiliations:** 1 Department of Medicinal Chemistry and Pharmacognosy, University of Illinois at Chicago, Chicago, Illinois, United States of America; 2 Department of Pharmaceutical Chemistry, Faculty of Pharmacy, Tanta University, Tanta, Egypt; 3 Department of Physiology and Biophysics, University of Illinois at Chicago, Chicago, Illinois, United States of America; University of Kansas Medical Center, UNITED STATES

## Abstract

The mechanism of action of histone deacetylase inhibitors (HDACi) is mainly attributed to the inhibition of the deacetylase catalytic activity for their histone substrates. In this study, we analyzed the abundance of class I HDACs in the cytosolic, nuclear soluble and chromatin bound cellular fractions in breast cancer cells after HDACi treatment. We found that potent *N*-hydroxy propenamide-based HDACi induced a concentration dependent decrease in the HDAC1 associated with chromatin and a lasting concomitant increase in cytoplasmic HDAC1 while maintaining total protein expression. No such change occurred with HDAC2 or 8, however, an increase in cytoplasmic non-phosphorylated HDAC3 was also observed. The subcellular re-equilibration of HDAC1 was subsequent to the accumulation of acetylated histones and might be cell cycle dependent. This study suggests that the biological activity of a subset of *N*-hydroxy propenamide-based HDACi may stem from direct competition with histone substrates of HDACs as well as from spatial separation from their substrates in the nucleus and/or change in post-translational modification status of HDACs.

## Introduction

Gene expression is controlled through modification of histone tails, which relaxes or condenses chromatin at different loci and effectively promotes or prevents access of the transcriptional machinery to DNA. Histone deacetylase (HDAC) is a family of 18 human enzymes that catalyze the removal of acetyl marks form histones and other protein substrates. HDACs are classified based on sequence homology to yeast and the classical zinc-dependent HDACs are class I–HDAC1, 2, 3 and 8 –class II–HDAC4, 5, 6, 7, 9 and 10 –and class IV–HDAC11 [1, 2]. Although HDAC11 closely relates to class I HDACs, the sequence similarity is too low to be classified as a class I HDAC [3]. Deacetylation of histones is mainly attributed to the class I HDACs, which has been shown to regulate a variety of cellular processes including cell cycle, proliferation, DNA repair, differentiation, and apoptosis [1, 4]. Given their role in gene regulation, HDACs have emerged as promising targets for treating various cancers and a range of autoimmune and neurodegenerative diseases, with four HDAC inhibitors (HDACi) FDA-approved to treat cutaneous or peripheral T-cell lymphoma or multiple myeloma [1].

**Fig 1:**
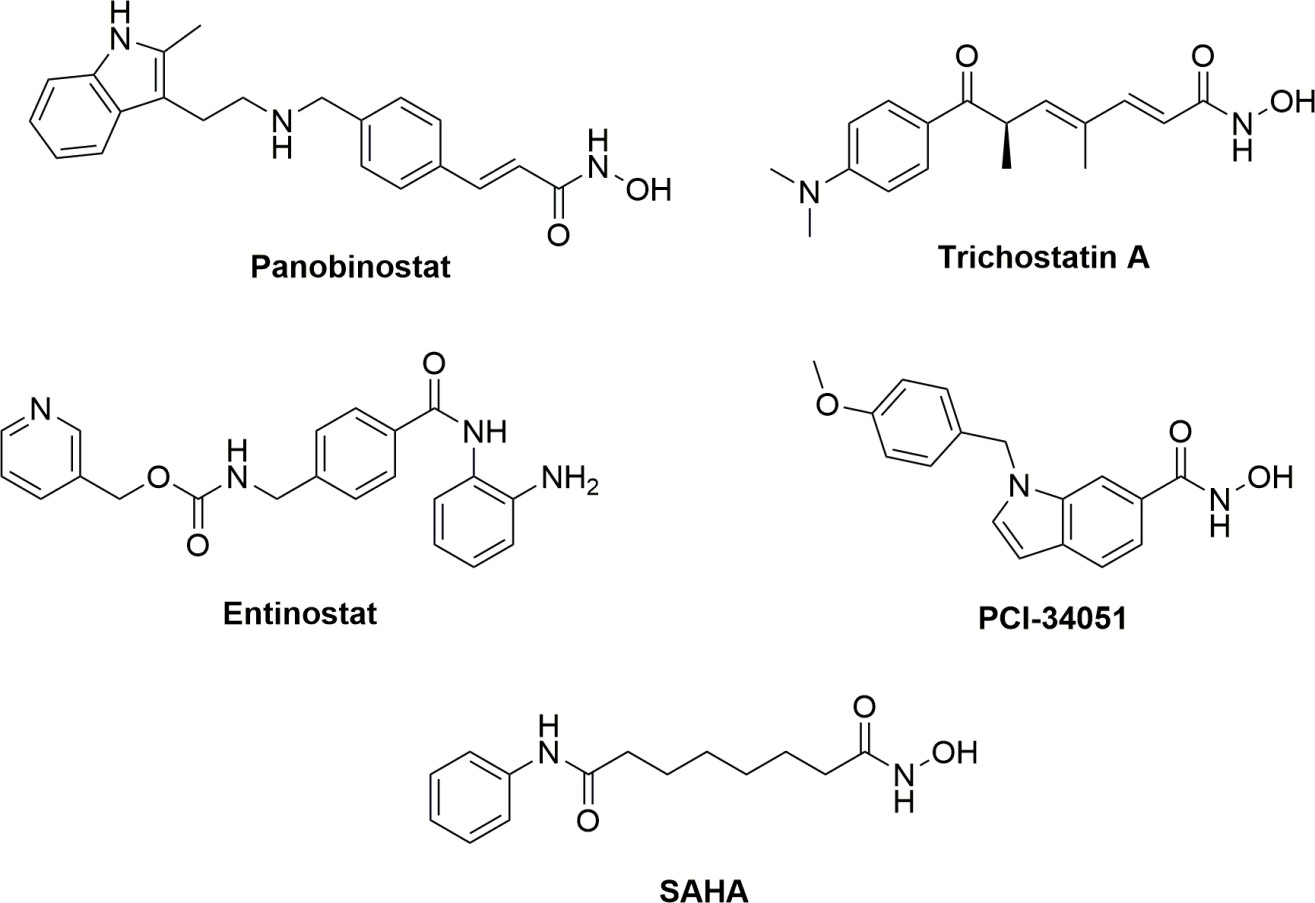
Structures of a diverse selection of HDACi. Panobinostat, trichostatin A, and SAHA are pan-isoform inhibitors. Entinostat is a class I selective inhibitor. PCI-34051 is an HDAC8 selective inhibitor.

In general, HDACi consist of 1) a zinc binding group, 2) surface binding group and 3) a linker to connect these two components and span the hydrophobic active site channel. Several structural classes with variation of these components exist, including long chain hydroxamic acids like suberoylanilide hydroxamic acid (SAHA, Fig 1), a pan inhibitor of class I and II HDACs; *N*-hydroxy propenamides such as panobinostat and trichostatin A (Fig 1), also pan class I and II inhibitors; “linkerless” hydroxamic acids such as PCI-34051 (Fig 1), an HDAC8 selective inhibitor; and ortho-aminoanilides such as entinostat (Fig 1), a class I selective inhibitor.

The effects of HDACi are mainly attributed to competitive inhibition of the deacetylase catalytic activity for their histone substrates. Under normal physiological conditions, HDAC catalytic activity is regulated through several mechanisms including subcellular localization and phosphorylation. In general, it has been shown that the class I HDACs reside in three separate pools: chromatin bound, soluble nuclear and cytosolic [5, 6]. HDAC1 and 2 are generally thought to be exclusively localized in the nucleus. Phosphorylation of HDAC1 and 2 promotes enzymatic activity and in the case of HDAC2 its association with chromatin [7]. HDAC3 has been shown to shuttle between the nucleus and cytosol, which is induced by cell cycle progression [8], DNA repair, [9] and differentiation [10]. Similar to HDAC1 and 2, HDAC3 phosphorylation also promotes enzymatic activity.

In this study, we investigated how HDACi affect the mechanisms that normally regulate the catalytic activity of class I HDACs in human breast cancer cell lines. MCF-7 and MDA-MB-231 cell lines were chosen as several HDACi have been shown to induce scaffold dependent effects on cell cycle and viability [11–14]. We found that the subcellular localization of select class I HDACs is dose dependently altered in response to a subset of HDACi scaffolds without changing the total cellular abundance. The re-equilibration of subcellular localization was only observed for HDAC1. For HDAC3, however, we observed a change in the phosphorylation state in response to treatment with HDACi. In addition, we noted that the re-equilibration of HDAC1 localization was subsequent to accumulation of histones and might be related to the cell cycle. This study adds a spatial and post-translational component to the mechanism of action of HDACi in conjunction to inhibition of catalytic activity.

## Materials and methods

### Cell culture and HDACi treatment

Human cell line MCF-7 was obtained from Dr. Debra Tonetti (University of Illinois at Chicago) and was maintained in RPMI supplemented with 10% FBS, 1% non-essential amino acids, 2 mM L-glutamine, 1% Pen-Strep and 0.01 mg/mL human recombinant insulin at 37°C in 5% CO_2_. MDA-MB-231 human cell line was obtained from Dr. Clodia Osipo (Loyola University Chicago) and was maintained in IMEM media (Corning) supplemented with 10% FBS, 1% non-essential amino acids, 2 mM L-glutamine, 10 mM HEPES and 1% Pen-Strep at 37°C in 5% CO_2_. Cells (2.1x10^6^) were plated in 10-cm plates in culture media for biochemical fractionation and whole cell lysate analysis. For confocal microscopy, cells were plated in an 8-well chambered slide (Thermo Fischer) at a density of 35,000 cells per well. After 48 hours, culture media was replaced with serum free media. After 24 hours, cells (80–90% confluence) were treated with HDACi (Selleckchem) at a concentration of 0.2, 10, or 50 μM in serum free medium. These concentrations span above and below all previously reported cell-based EC_50_ [11, 12, 15, 16]. For cell cycle analysis, cells were seeded in a clear-bottom 96-well plate, serum starved for 24 hours followed by HDACi treatment for 12 hours. The cells were then fixed with ice-cold methanol for 15 minutes, stained with propidium iodide for 40 minutes and imaged with Celigo image cytometer (Nexcelom Bioscience). Cell cycle analysis was conducted using FCS Express 6 Multicycle application (De Novo Software).

### Biochemical cellular fractionation

Our method for biochemical fractionation was developed based on a previous work [17]. Briefly, cells were washed with phosphate buffered saline (PBS) and then scraped with a rubber policeman in PBS. Cells were then pelleted by centrifugation at 1,000g for 5 minutes at 4°C in a 1.7 mL Eppendorf tube (Thermo Fischer). The pellet was then resuspended in 300 μL ice-cold cytosolic lysis buffer (10 mM HEPES, 10 mM KCl, Igepal CA-630 (Sigma-Aldrich, 0.2% for MCF-7 cells and 0.05% for MDA-MB-231 cells), 1X cOmplete Protease Inhibitor Cocktail (Roche), and 1:100 Phosphatase Inhibitor Cocktail Set II (EMD Millipore)) and vortexed for 10 seconds at room temperature (RT). The cells were allowed to swell for 10 minutes at RT and then vortexed again for 10 seconds at RT. Next, the nuclei were pelleted by centrifugation at 6,500g for 5 minutes at 4°C, the supernatant (cytosolic fraction) was collected and the nuclear pellet was washed with ice-cold cytosolic lysis buffer without Igepal CA-630. The nuclei were again pelleted at 6,500g for 5 minutes at 4°C and the supernatant discarded. The nuclear pellet was resuspended in 150 μL ice-cold no salt lysis buffer (3 mM EDTA, 1X cOmplete Protease Inhibitor Cocktail, and 1:100 Phosphatase Inhibitor Cocktail Set II) by sonication (5 seconds) on ice. The solution was then incubated with rotation for 30 minutes at 4°C. Chromatin was pelleted by centrifugation at 6,500g for 5 minutes at 4°C, the supernatant (nuclear soluble fraction) was collected and chromatin was resuspended in ice-cold high salt lysis buffer (50 mM HEPES, 0.5 M NaCl, 0.05% Igepal CA-630, 1X cOmplete Protease Inhibitor Cocktail, and 1:100 Phosphatase Inhibitor Cocktail Set II) by vortexing for 2 minutes in 30-second rounds at RT. The solution was then incubated with rotation for 30 minutes at 4°C. The DNA and nuclear matrix were pelleted by centrifugation at 14,000g for 10 minutes at 4°C. The supernatant (chromatin bound fraction) was collected and the pellet was discarded. The chromatin bound fraction was dialyzed into a lower salt concentration buffer (10 mM HEPES, 100 mM NaCl, 10 mM KCl, 5% glycerol, and 0.3% Igepal CA-630, 1X cOmplete Protease Inhibitor Cocktail, and 1:100 Phosphatase Inhibitor Cocktail Set II) to improve SDS-PAGE running conditions as described previously [18]. All fractions were kept on ice until further analysis and then stored at -20°C.

### Whole cell lysate preparation

Cells were washed with PBS, scraped with a rubber policeman in PBS and pelleted by centrifugation at 1,000g for 5 minutes at 4°C in a 1.7 mL Eppendorf tube. Next, the pellet was resuspended in 300 μL ice-cold RIPA lysis buffer (150 mM NaCl, 0.5% sodium deoxycholate, 0.1% sodium dodecyl sulfate, 50 mM Tris pH 8.0, 1% Igepal CA-630, 1X cOmplete Protease Inhibitor Cocktail, and 1:100 Phosphatase Inhibitor Cocktail Set II) and incubated with rotation for 30 minutes at 4°C. Insoluble cellular matrix was pelleted at 14,000g for 10 min at 4°C and the supernatant was collected and kept on ice until further analysis and then stored at -20°C.

### Western blotting

Protein concentration of each cellular fraction was characterized with the Pierce BCA Assay Kit (Thermo Fischer). Proteins were diluted 3:1 with 4X Laemmli sample buffer containing 5% β-mercaptoethanol (Bio-Rad), boiled for 5 minutes, loaded onto a 12% polyacrylamide gel and electrophoretically separated (100 V, 1.5 hours). Equal portions of cytosolic, nuclear soluble and chromatin bound fractions were used for each experiment. After electrophoresis, proteins were transferred to a nitrocellulose membrane (iBlot 2 (Invitrogen) mode P3 for 7 minutes). Equal protein loading was confirmed with Ponceau S staining (Sigma-Aldrich). After staining, the membranes were blocked with Odyssey blocking buffer (Li-Cor) for 2 hours at RT, and then incubated with primary antibodies in blocking buffer for HDAC1 (Abcam, ab7028, lot GR188529-1, rabbit), HDAC2 (Abcam, ab124974, lot GR97402-7, rabbit), HDAC3 (Abcam, ab7030, lot GR121157, rabbit), non-phosphorylated HDAC3 (Ref [19]; Millipore, 05–813, lot 2726719, mouse), GAPDH (Abcam, ab128915, lot GR90965-22, rabbit), TATA-binding protein (Abcam, ab818, lot GR131329-14, mouse), H3 (Abcam, ab1791, lot GR242835-1, rabbit) and Acetyl-histone H3 (Millipore, 06–599, lot 2153150, rabbit) overnight at 4°C. The membranes were then washed three times with PBS containing 0.1% Tween-20 (PBST) for 5 minutes at RT. The membranes were then incubated with an anti-rabbit or anti-mouse IRDye-conjugated secondary antibody (Li-Cor) for 1 hour at RT. The membranes were then washed 3 times with PBST for 5 minutes and visualized using the Odyssey Sa scanner (Li-Cor). Densitometry analysis was performed with Image Studio version 5.2 (Li-Cor).

### Confocal microscopy

Cells were washed two times with PBS and fixed by incubating with 4% formaldehyde (Sigma-Aldrich) in PBS for 10 minutes at RT. Cells were then washed two times with PBS for 5 minutes at RT. Next, the cells were permeabilized by incubating with 0.1% Triton X-100 (Thermo Fischer) in PBS for 1 min at RT. After permeabilization, the cells were washed two times with PBS and then blocked with 10% goat serum (Thermo Fischer) for 1 hour at RT. The blocking buffer was decanted and rabbit monoclonal HDAC1 antibody (Abcam) in blocking buffer was added. After incubation at 4°C overnight, the cells were washed two times with PBS for 5 minutes at RT and then incubated for 1 hour at RT with Alexa Flour 488-conjugated goat anti-rabbit secondary antibody (Thermo Fischer) in 1% goat serum (Thermo Fischer). The cells were then washed two times with PBS for 5 minutes at RT, dried for 5 minutes, mounted with Prolong Gold Antifade Mountant containing DAPI (Thermo Fischer) and allowed to cure in the dark for 24 hours. The slide was visualized with a Zeiss LSM 710 (25 mW Multi-line Ar laser for Alexa Flour 488, 30 mW diode UV laser for DAPI) containing a 63x/1.46 Oil alpha Plan-Apochromat objective.

The correlation between the HDAC1 fluorescence signal and the DAPI stain signal was analyzed with JACoP (ImageJ) and the Pearson’s coefficient calculated for each HDACi optical section. Pearson’s coefficient range from 1 to −1, with 1 standing for complete positive correlation and −1 for a negative correlation, and zero standing for no correlation.

### Statistical analysis

Statistical analyses were performed with GraphPad Prism 7 software. All data are shown as mean ± standard deviation. Student's t-test (two-tailed) was used to measure statistically significant differences between groups. P value < 0.01 was considered statistically significant for this study.

## Results

### HDACi affect the subcellular localization of HDAC1

**Fig 2:**
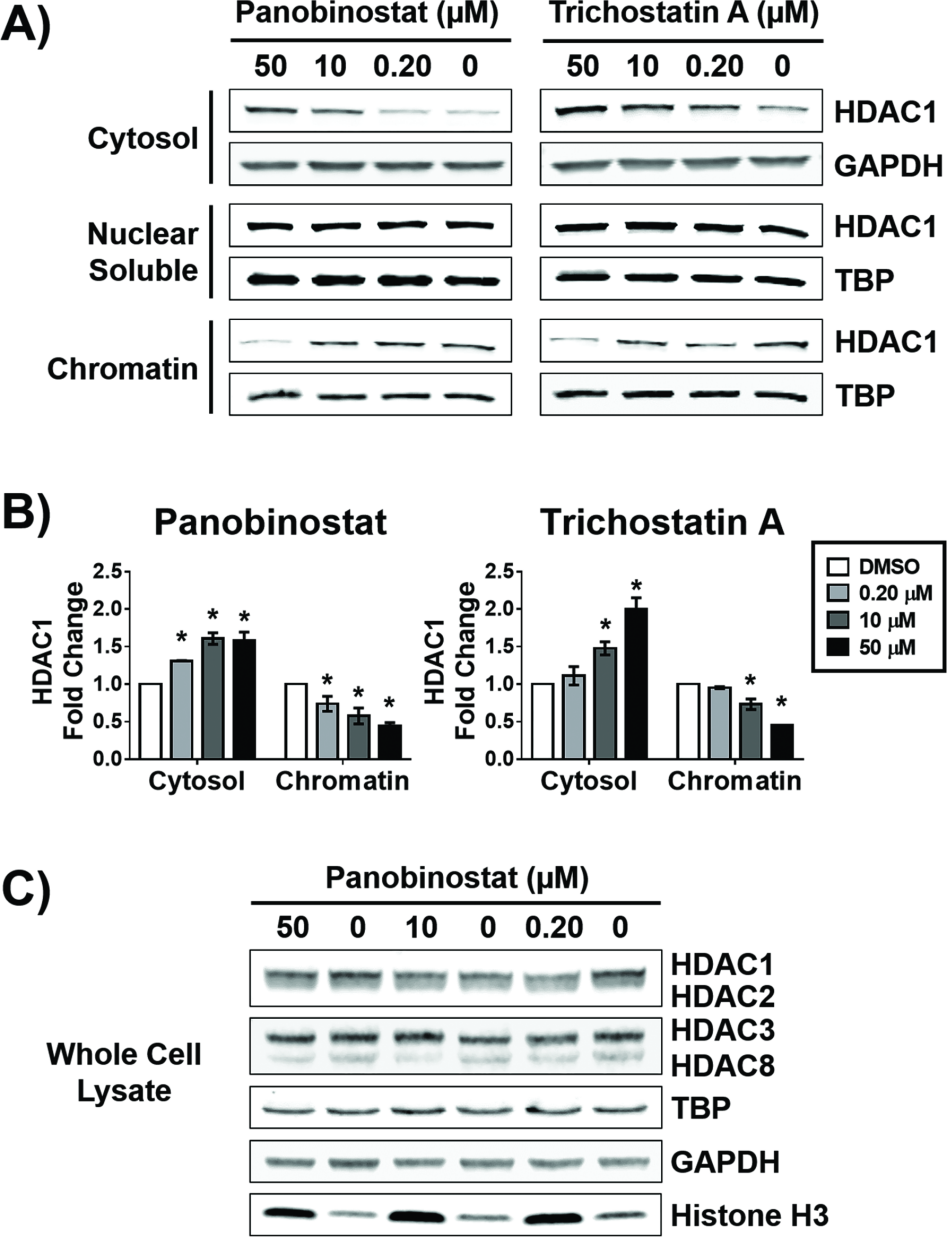
Potent HDACi alter the subcellular localization of HDAC1. MCF-7 cells were treated with indicated concentrations of panobinostat or trichostatin A for 12 hours. A) Western blot analysis of the abundance of HDAC1 in the cytosolic, nuclear soluble, and chromatin bound fractions. B) Densitometry analysis of the abundance of HDAC1 normalized to GAPDH (cytosolic fraction) or to TATA-binding protein (TBP, nuclear soluble and chromatin bound fractions). C) Western blot analysis of the total abundance of class I HDACs and the loading controls TBP, GAPDH, and histone H3 after treatment with indicated concentrations of panobinostat for 12 hours. * Statistically significant difference compared with DMSO control (Student’s t-test, P<0.01). Western blots shown are representative of at least two independent experiments. HDAC1 fold change is presented as the mean of at least two independent experiments ± standard deviation.

As three separate pools of HDACs, cytosolic, nuclear soluble, and chromatin bound fractions have been previously reported [5, 6], we sought to analyze the effect that HDAC inhibition may have on the abundance of HDACs in these fractions. We treated serum starved MCF-7 cells with five structurally diverse pan and isoform selective HDACi (Fig 1) at 0.2, 10, and 50 μM for 12 hours followed by biochemical fractionation of cells into cytosolic, nuclear soluble, and chromatin bound portions. These concentrations of inhibitors were chosen as they span both above and below the reported cell-based EC_50_’s [11, 12, 15, 16] and *in vivo* plasma concentrations [20–24] for all the compounds evaluated in this study. We treated cells aligned at G_0_/G_1_ with HDACi so that only one cell cycle was analyzed, as the doubling time of MCF-7 cells is 24 hours [25]. Western blot analysis of these fractions shows that 12-hour treatment with panobinostat and trichostatin A, but not SAHA, entinostat or PCI-34051, induce a statistically significant concentration-dependent decrease of the chromatin bound HDAC1 fraction and a concomitant increase in the cytoplasmic fraction (Fig 2A and 2B, S1 Fig). This was exclusive for HDAC1, in comparison to the other class I HDACs 2, 3 or 8 (S1 Fig).

At 0.2, 10, and 50 μM, panobinostat reduced HDAC1 bound to chromatin to 74±10, 58±11 and 44±5.0 percent of the DMSO control, respectively. At the same concentrations, trichostatin A reduced the HDAC1 bound to chromatin to 95±1.5, 73±7.0 and 45±0.048 percent of the DMSO control, respectively. Both panobinostat and trichostatin A did not significantly affect cell viability compared with DMSO control (S2 Fig). Neither of the HDACi affected the abundance of HDAC1 in the nuclear soluble cellular fraction (Fig 2A). Similar to MCF-7 cells, we also observed trichostatin A, but not entinostat affected the subcellular distribution of HDAC1 in another cell line, MDA-MB-231 (S3 Fig). To investigate whether the total abundance of HDAC1 was changing in response to the HDACi that were affecting its subcellular localization, we prepared whole cell lysates from cells treated with 0.2, 10, and 50 μM panobinostat. We observed no difference in the total abundance of HDAC1, HDAC2, 3 and 8 at any concentration of panobinostat in comparison to DMSO control (Fig 2C). The validity of the biochemical fractionation was confirmed by the absence of GAPDH and HDAC8 [26–28] in the nuclear soluble and chromatin bound cellular fractions, and the absence of TATA-binding protein (TBP) in the cytosolic cellular fraction (data not shown). Moreover, HDACi treatment did not alter the total cellular abundance of these loading controls (Fig 2C). We initially attempted to use histone H3 (H3) as the nuclear loading control, however, we observed that treatment with panobinostat increased its abundance in comparison to DMSO (Fig 2C).

**Fig 3:**
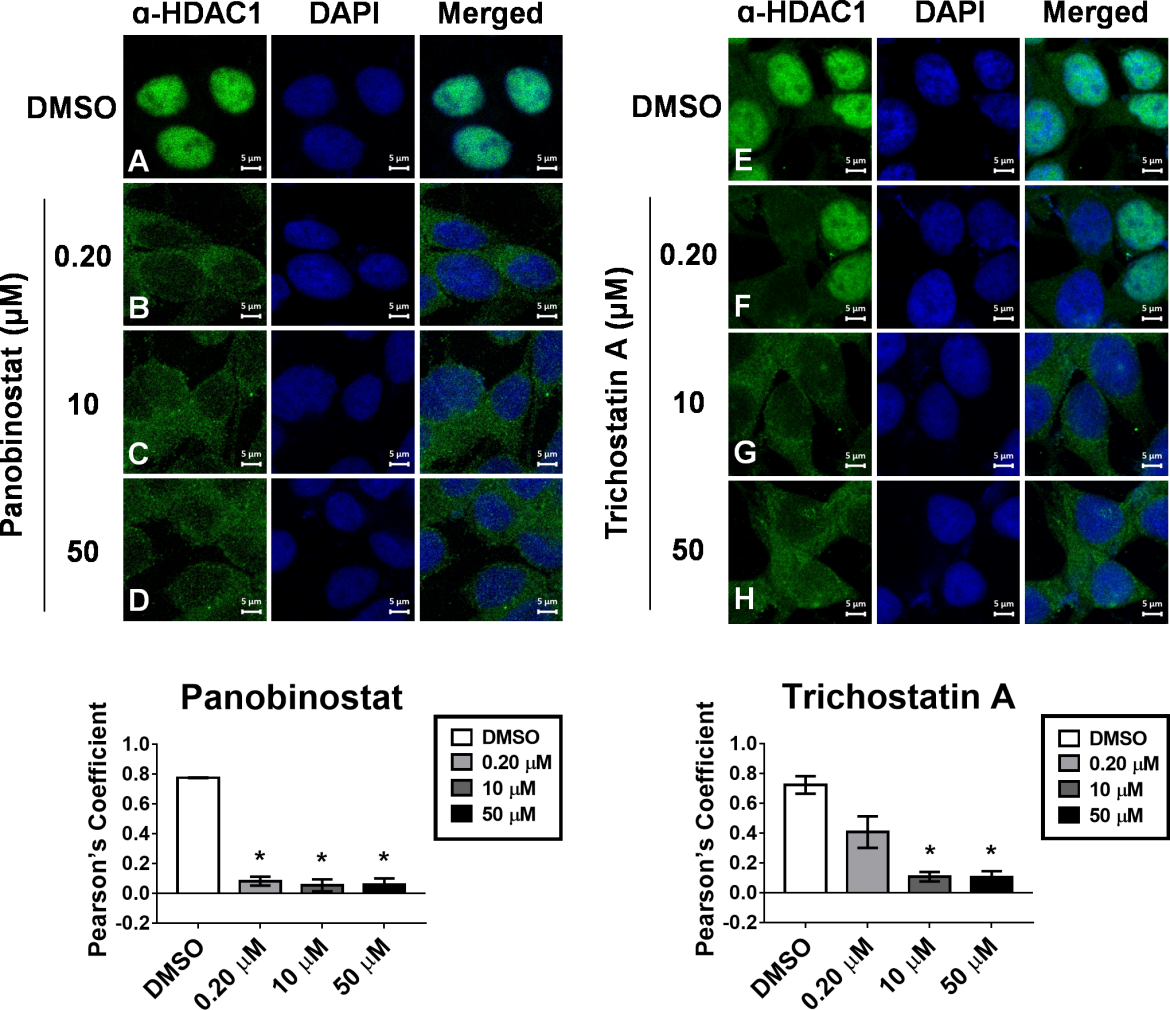
HDACi-induced re-equilibration of HDAC1 is confirmed by confocal microscopy. MCF-7 cells were treated with indicated concentrations of panobinostat (optical sections A-D, respectively) or trichostatin A (optical sections E-H, respectively) for 12 hours, fixed, permeabilized and optical sections were obtained by laser scanning confocal microscopy. Fluorescence signal for HDAC1 is shown in green (left panels), DAPI staining is shown in blue (middle panels), and merged optical sections are shown in the right panels. Colocalization analysis of HDAC1 fluorescence signal and the DAPI stain signal was performed with JACoP (ImageJ) and shown below. * Statistically significant difference compared with DMSO control (Student’s t-test, P<0.01). Pearson’s Coefficient is presented as the mean of at least two independent experiments ± standard deviation. Optical sections shown are representatives of at least two independent experiments.

To confirm the HDACi-induced change in HDAC1 subcellular distribution in an orthogonal assay, we prepared MCF-7 cell microscope slides treated with HDACi under the same conditions as those for the biochemical fractionation. Optical sections were obtained for each HDACi by laser scanning confocal microscopy (Fig 3, S4 Fig) and correlated well with the results obtained by biochemical fractionation for HDAC1 (Fig 2A and 2B, S1 Fig). HDAC1 colocalized with nuclear DAPI staining in the DMSO-treated cells for all experiments (Fig 3, optical sections A, E), whereas it localized in the cytosol when treated with panobinostat or trichostatin A (Fig 3, optical sections B-D and F-H, respectively). Both results were confirmed by quantitative colocalization analysis (Fig 3). The cells treated with SAHA or entinostat did not exhibit any significant change in subcellular localization of HDAC1 even at 50 μM, the highest concentration tested, (S4 Fig, optical sections B-D and F-H, respectively) and were similar to the DMSO-treated cells (S4 Fig, optical sections A and E, respectively). Cells treated with PCI-34051 did not exhibit any change in subcellular localization of HDAC1 at 0.2 or 10 μM in comparison to DMSO control (S4 Fig, optical sections I-K). At 50 μM PCI-34051, cells became elongated in morphology and did not adhere to the glass slide preventing reproducible staining (S4 Fig, optical section L).

### Re-equilibration of HDAC1 subcellular localization is subsequent to accumulation of acetylated histones and is affected by mitogens

**Fig 4:**
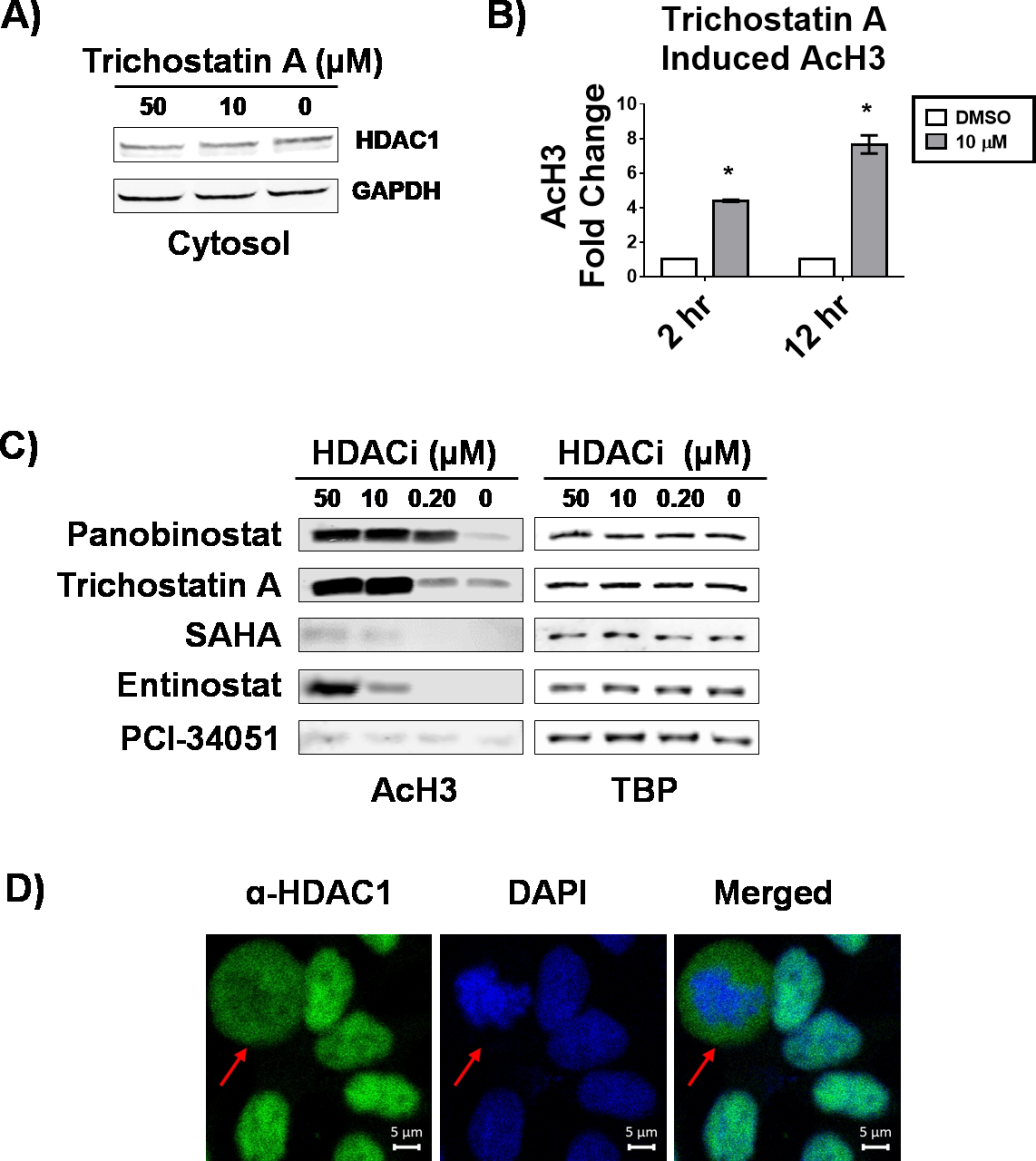
HDAC1 re-equilibration induced by HDACi is subsequent to histone acetylation and is affected by mitogenic stimuli. A) Western blot analysis of cytosolic fractions of MCF-7 cells treated with indicated concentrations of trichostatin A for 2 hours. B) Densitometry analysis of western blots of chromatin fractions from MCF-7 cells treated with 10 μM trichostatin A for 2 and 12 hours; change in AcH3 was normalized to TBP. C) Western blot analysis of chromatin bound fractions from MCF-7 cells treated with indicated concentrations of HDACi for 12 hours. D) Laser scanning confocal microscopy of MCF-7 cells, grown with 10% serum and treated with DMSO for 12 hours. Fluorescence signal for HDAC1 is shown in green (left panels), DAPI staining is shown in blue (middle panels), and merged optical sections are shown in the right panels. Representative optical section from two independent experiments is shown. Arrows indicate mitotic cells where HDAC1 is dispersed off chromatin, as indicated by DAPI staining. * Statistically significant difference compared with DMSO control (Student’s t-test, P<0.01).

The efficacy of HDACi is generally attributed to changing gene expression by leading to an accumulation of acetylated histones. As we have observed a change in the subcellular localization of HDAC1 induced by HDACi, we wondered whether this precedes or is subsequent to accumulation of histone acetylation. We tested this hypothesis by comparing histone acetylation after treatment with 10 μM trichostatin A for two and 12 hours, and compared to the abundance of HDAC1 in the cytosol (Fig 4A and 4B). We used 10 μM trichostatin A as this concentration robustly affected subcellular localization of HDAC1 (Fig 2A and 2B). At the two-hour time point, there was an accumulation of total acetylation of H3, a known HDAC1 substrate, whereas no increase in cytosolic HDAC1 was observed, suggesting acetylation precedes accumulation of HDAC1 in the cytosol (Fig 4A and 4B). We also analyzed the change in acetylation of H3 in response to 12-hour treatment with 0.2, 10, and 50 μM trichostatin A or the other four HDACi used in this study (Fig 4C). We found change in H3 acetylation correlated well with the previously reported in vitro IC_50_ values for each of the compounds [13]. However, the change in H3 acetylation did not correlate with the compounds’ ability to change HDAC1 subcellular localization, given entinostat showed a large increase in H3 acetylation, but did not affect HDAC1 subcellular localization. Taken together, the in vitro HDAC inhibitor activity of the compounds reported previously ([12, 13, 29, 30]) and the increase in histone acetylation rule out cell permeability as the reason for the differences these compounds impose on HDAC1 subcellular localization.

As others have noted a cell cycle dependent association of HDAC1 with chromatin [7, 31], our experimental investigation into its subcellular localization after treatment with HDACi was initially conducted after serum starvation (without mitogens). In addition, we obtained optical sections of cycling MCF-7 cells treated with 0.2, 10, and 50 μM panobinostat for 12 hours (data not shown). Unlike our experiments with quiescent cells, HDAC1 localization in DMSO controls depended on cell cycle (Fig 4D), with HDAC1 diffusing from chromatin during mitosis, confounding interpretation of samples treated with HDACi.

### Re-equilibration of HDAC1 subcellular localization is sustained after removal of HDACi

**Fig 5:**
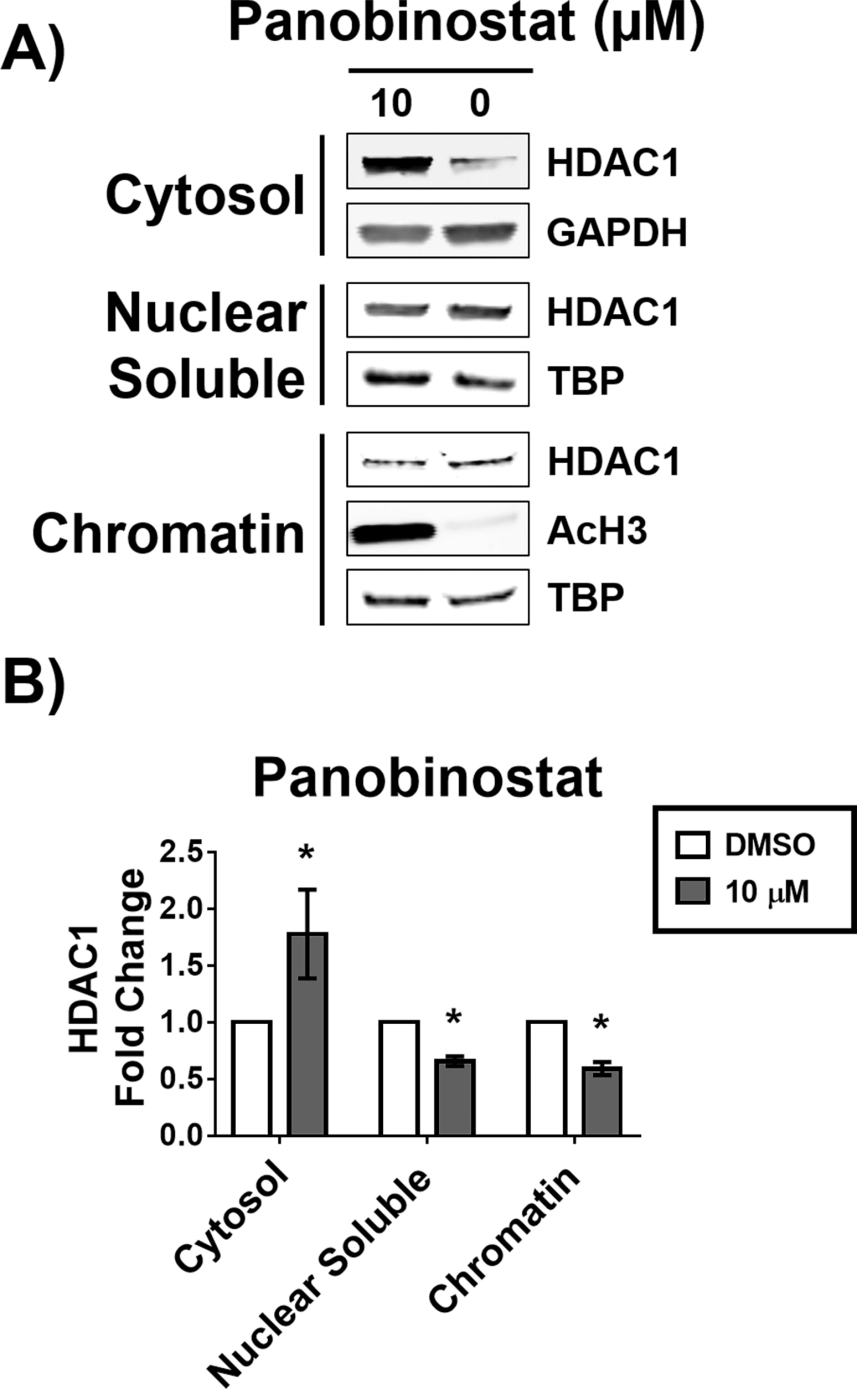
Increase in cytosolic HDAC1 is irreversible up to 24 hours. MCF-7 cells were treated with 10 μM panobinostat for 12 hours, the compound was then removed and cells allowed to recover for 24 hours. A) Western blot analysis of the abundance of HDAC1 in the cytosolic, nuclear soluble, and chromatin bound fractions. B) Densitometry analysis of the abundance of HDAC1 normalized to GAPDH (cytosolic fraction) or to TATA-binding protein (TBP, nuclear soluble and chromatin bound fractions). * Statistically significant difference compared with DMSO control (Student’s t-test, P<0.01). Western blots shown are representative of at least two independent experiments. HDAC1 fold change is presented as the mean of at least two independent experiments ± standard deviation.

As we saw a large percentage, up to 60%, decrease of the HDAC1 associated with chromatin upon treatment with panobinostat, we questioned whether this was a sustained change, or whether the cell could recover. To answer this question, we pulsed MCF-7 cells with 10 μM panobinostat treatment for 12 hours, and allowed the cells to recover for approximately one cell cycle (24 hours) in fresh media without the inhibitor. We observed that the cytosolic fraction of HDAC1 remained increased, and the chromatin bound fraction remained decreased in response to treatment with panobinostat (Fig 5A and 5B). In addition, the nuclear soluble fraction of HDAC1 decreased (Fig 5A and 5B), which did not occur with 12-hour treatment of panobinostat (Fig 2A). Furthermore, H3 acetylation remained increased similar to 12-hour treatment with panobinostat (Fig 4B).

### HDAC3 phosphorylation state is affected by HDACi treatment

**Fig 6:**
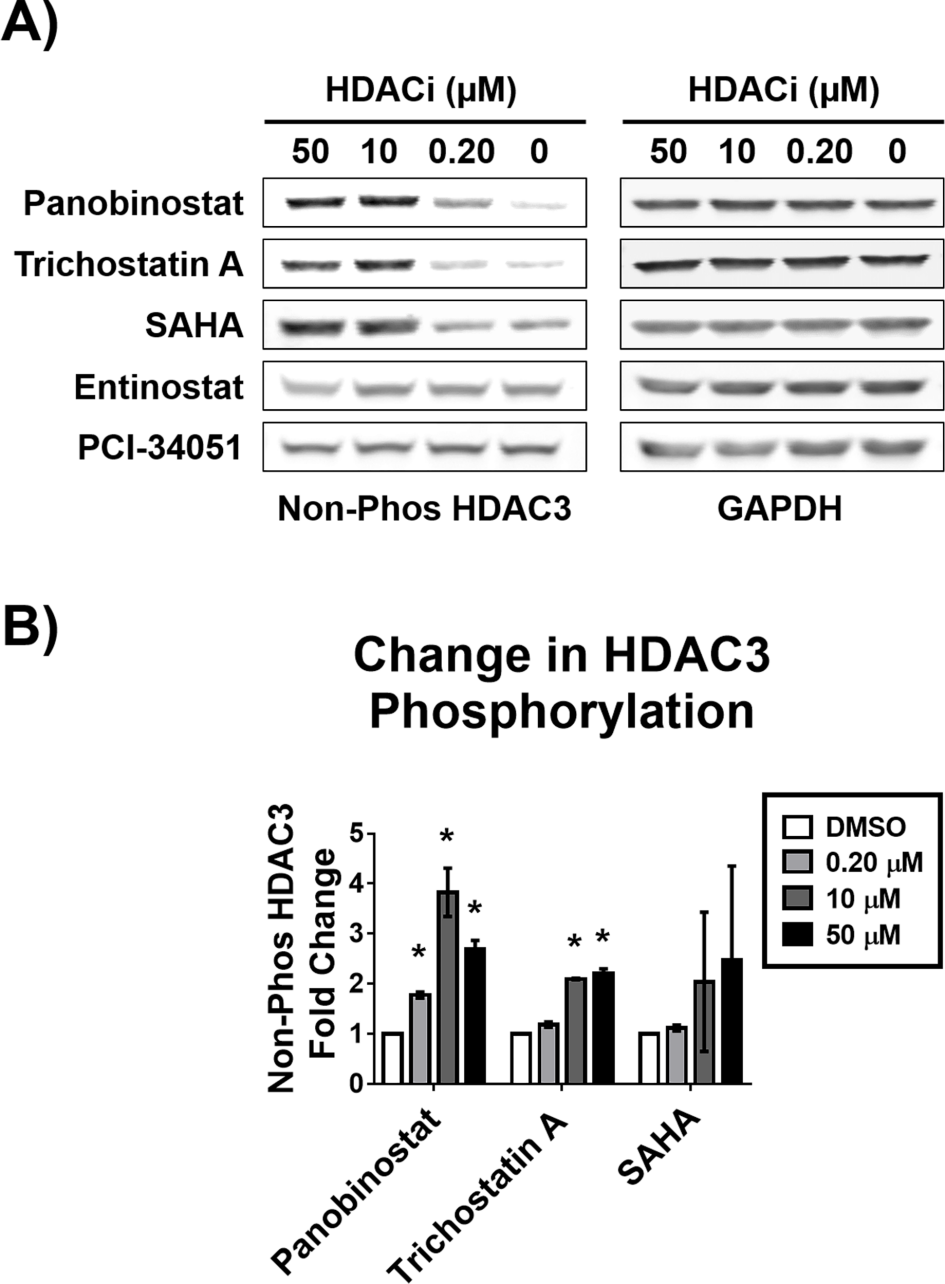
Potent HDACi increase the abundance of non-phosphorylated HDAC3. MCF-7 cells were treated with indicated concentrations of panobinostat, trichostatin A, or SAHA for 12 hours and then biochemically fractionated. A) The abundance of non-phosphorylated HDAC3 was characterized by Western blot analysis in the cytosolic fraction. B) Densitometry analysis of the abundance of non-phosphorylated HDAC3 normalized to GAPDH. * Statistically significant difference compared with DMSO control (Student’s t-test, P<0.01). Western blots shown are representative of at least two independent experiments. HDAC3 fold change is presented as the mean of at least two independent experiments ± standard deviation.

**Fig 7:**
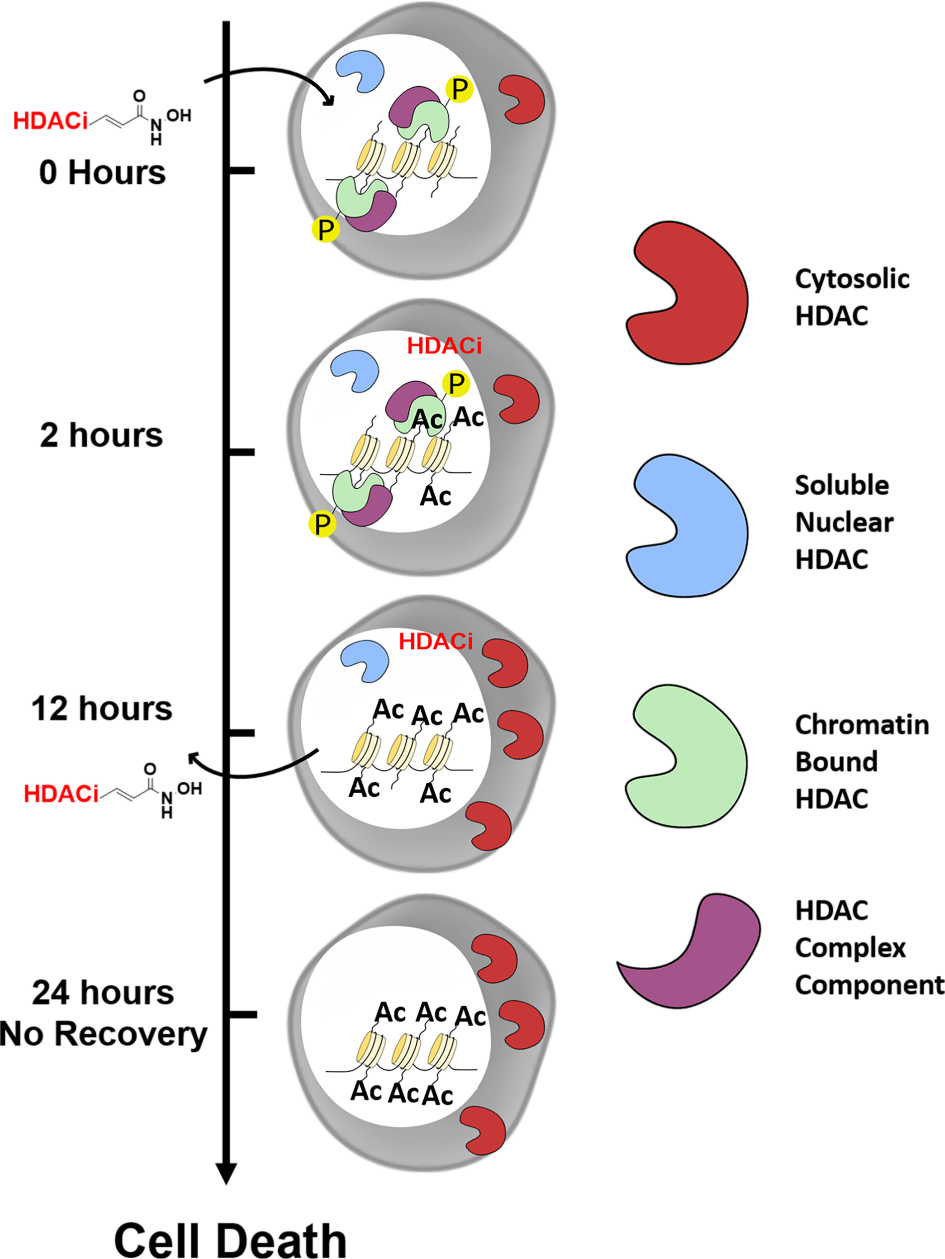
A model of the mechanism of action of propenamide-based HDACi including re-equilibration of the subcellular distribution and modulation of the post-translational modification of HDACs. Treatment of with *N*-hydroxy propenamide-based HDACi increase histone acetylation at 2 hours without affecting the subcellular localization or the phosphorylation status of HDACs. At 12 hours, re-equilibration of subcellular localization as well as a decrease in phosphorylation of HDACs is observed, along with a greater increase in histone acetylation. The induced change in subcellular localization and phosphorylation of HDACs is sustained 24 hours after removal of HDACi. This is in line with the time necessary to induce commitment to cell death with HDACi.

In addition to subcellular localization, we also investigated HDAC3 phosphorylation state in response to HDACi treatment, as this isoform’s subcellular localization was not affected (S1 Fig). Using an antibody found to be specific for non-phosphorylated HDAC3 [19], we analyzed the amount of non-phosphorylated HDAC3 after treatment with 0.2, 10 and 50 μM panobinostat, trichostatin A, SAHA, entinostat or PCI-34051 (Fig 6A). We observed 1.8–3.8 and 1.8–2.3-fold increase in non-phosphorylated HDAC3 in response to panobinostat and trichostatin A, respectively (Fig 6B). We found SAHA, entinostat, and PCI-34051 did not induce a significant change in non-phosphorylated HDAC3 (Fig 6A and 6B). These results along with the HDACi-induced re-equilibration of the subcellular localization are summarized in Fig 7.

## Discussion

HDAC catalytic activity is normally regulated by controlling subcellular localization and phosphorylation status, among other mechanisms. In this study, we found that HDACi, whose effects are generally thought to be associated with competition with endogenous histone substrates, also affect subcellular localization and phosphorylation of class I HDACs in a chemotype dependent manner.

In particular, the pan HDACi panobinostat and trichostatin A induced a dose dependent increase in the cytosolic HDAC1 and a decrease in chromatin bound HDAC1 in MCF-7 and MDA-MB-231 cells. This response was not detected for the other class I HDACs. Instead, we observed a change in the phosphorylation state of HDAC3. Both panobinostat and trichostatin A dose dependently increased non-phosphorylated HDAC3 in the cytoplasm, with no change in total HDAC3 expression. Given that the total HDAC1 expression was not affected as well, the change in HDAC1 subcellular localization could either be caused by a translocation from chromatin to cytosol, or a degradation of chromatin bound HDAC1 coupled with a matching increase in translation of HDAC1 in the cytoplasm. HDACs are key regulators of gene expression, and the set of HDACi used in this study differentially affects gene expression in breast cancer cell lines used [15, 32–35]. Others have also observed that trichostatin A treatment increases HDAC1 mRNA levels [36]. As cells respond to trichostatin A by increasing HDAC1 mRNA production, a degradation/increased translation mechanism is likely responsible for our observation, which warrants future validation by blocking protein synthesis with cycloheximide or actinomycin D. The re-equilibration of HDAC1 subcellular localization likely play a role in the mechanism of action of HDACi given that it was significant starting at 200 nM and 10 μM for panobinostat and trichostatin A, respectively, which are within concentrations reported *in vitro* and *in vivo* [15, 16, 20–22]. The observations appear to be independent of the estrogen receptor (ER) status considering that they were observed in ER+ MCF-7 and ER- MDA-MB-231 cells.

In addition, we observed that the re-equilibration of HDAC1 subcellular localization is sensitive to mitogenic stimuli. Re-equilibration of HDAC1 subcellular localization induced by panobinostat could be clearly observed by confocal microscopy only after cells were serum starved. When cells were cultured in the presence of serum, HDAC1 subcellular localization varied greatly in the DMSO control, depending on what part of the cell cycle an individual cell was in. Others have observed mitogens can induce HDAC1 dissociation from chromatin [37] and cell cycle dependent histone hyperacetylation has been noted going from G_1_ to S phase [37] and hypoacetylation leading into mitosis [31]. In our study, we found that histone hyperacetylation precedes re-equilibration of HDAC1 subcellular localization. Taken together these data suggest that HDACi, initially induce histone hyperacetylation, which may be recognized by the cell as a G_1_ to S phase transition, inducing a shift of HDAC1 from chromatin to cytosol, as would occur during normal cell cycle progression. As HDAC1 remains in the cytosol, the cell cannot progress from G_2_ to M phase when deacetylation is necessary for chromatin condensation, leading to arrest, which is in line with the observed G_2_/M arrest induced by HDACi (data not shown).

Curiously, we observed a sustained increase in cytosolic and decrease in chromatin bound HDAC1 after the pulse treatment with panobinostat followed by recovery for 24 hours, roughly one cell cycle. In addition, acetylation of H3 remained increased after 24-hour recovery. This model is depicted in Fig 7. Longer recovery times after pulse treatment of HDACi may yield a return to basal HDAC1 levels in the cytosol; however, pulse treatment with panobinostat has been shown to induce cell death starting around 12 hours [13], which is the same time necessary to induce re-equilibration of HDAC1 subcellular localization. Therefore, re-equilibration of HDAC1 subcellular localization may be irreversible and may play role in HDACi induced cell death, given their similar kinetics. Others have observed inhibitor binding and histone acetylation kinetics do not correlate well with the time to induce cell death [38]. Our study identifies a plausible additional component of the mechanism of action of HDACi that may better correlate with induction of cell death. This mechanism may be useful in drug discovery efforts since this subset of HDACi is associated with increased efficacy in a number of cancer cell types [13].

The re-equilibration of HDAC1 subcellular distribution and change in HDAC3 phosphorylation was HDACi scaffold dependent. We only observed significant changes in either of these phenomena with panobinostat and trichostatin A, but not SAHA, entinostat or PCI-34051 treatment. Both trichostatin A and panobinostat feature alkenyl groups in the linker portion which have been shown to affect the tertiary structure of targeted HDACs differently than compounds with alkyl linkers like SAHA [39]. Changes in tertiary structure could potentially affect HDAC protein-protein interactions, which is known to control HDAC subcellular localization [40]. Indeed, others have shown that trichostatin A disrupts HDAC1 complex formation more potently than SAHA [41].

The changes in subcellular localization or phosphorylation were not dependent upon the relative induction of histone H3 acetylation. While panobinostat and trichostatin A, caused the largest increase in H3 acetylation and the most significant change in HDAC1 subcellular localization or HDAC3 phosphorylation, SAHA and entinostat also induced H3 acetylation without significantly affecting HDAC1 subcellular localization. No change in histone acetylation was observed with PCI-34051 treatment, which is in line with reports showing it is an HDAC8 selective inhibitor; HDAC8 cannot deacetylate histones as we found it exclusively localized in the cytosol. Taken together our data show that only *N*-hydroxy propenamide-based HDACi scaffolds induce the change in HDAC subcellular localization or phosphorylation.

A large body of research has focused on the selective recruitment of HDACs to individual genes, to determine whether the gene is epigenetically silenced in a context of interest. These studies often employ chromatin immunoprecipitation (ChIP) and are typically validated with use of an HDACi to show that the HDAC activity is necessary for changing the expression of the gene. This study clearly demonstrates that some HDACi induce up to 60% dissociation of the total HDAC1 bound to chromatin, which should be taken into consideration when interpreting ChIP data.

This study suggests that the biological activity of a subset of *N*-hydroxy propenamide-based HDACi may stem from direct competition with histone substrates of HDACs as well as from spatial separation from their substrates in the nucleus and/or change in post-translational modification status of HDACs. Future studies are needed to investigate other HDACi chemotypes, elucidate the mechanism, expand beyond breast cancer cells, and determine if the same phenomenon is observed in vivo.

## Supporting information

S1 Fig The impact of HDACi treatment on the subcellular localization of class I HDACs. MCF-7 cells were treated with indicated concentrations of panobinostat, trichostatin A, SAHA, Entinostat, or PCI-34051 for 12 hours and then fractionated biochemically. The abundance of class I HDACs was characterized by Western blot analysis in the cytosolic (top panel), nuclear soluble (middle panel), and chromatin bound (bottom panel) fractions. Western blots shown are representative of at least two independent experiments.
(TIF)

S2 Fig HDACi treatment does not significantly affect MCF-7 cell viability. MCF-7 cells were serum starved for 12 hours and treated with indicated concentrations of panobinostat or trichostatin A. Cells were fixed, stained with propidium iodide (PI), and cell cycle analysis was conducted with Celigo image cytometer. Three-dimensional plot on left shows integrated PI intensity and table on right shows percentage of apoptotic cell population for each treatment. Percentage values are expressed as mean ± standard deviation of three replicates.
(TIF)

S3 Fig Trichostatin A treatment induces re-equilibration of HDAC1 subcellular localization in MDA-MB-231 cells. MDA-MB-231 cells were treated with 50 μM trichostatin A or entinostat for 12 hours and then fractionated biochemically. A) The abundance of HDAC1 was characterized by Western blot analysis in the cytosolic (top panel), and chromatin bound (bottom panel) fractions. B) Densitometry analysis of the abundance of HDAC1 normalized to GAPDH loading control.
(TIF)

S4 Fig HDACi-induced re-equilibration of HDAC1 analysis by confocal microscopy. MCF-7 cells were treated indicated concentrations of SAHA (optical sections A-D, respectively), entinostat (optical sections E-H, respectively), or PCI-34051 (optical sections I-L, respectively) for 12 hours, fixed, permeabilized and optical sections were obtained by laser scanning confocal microscopy. Fluorescence signal for HDAC1 is shown in green (left panels), DAPI staining is shown in blue (middle panels), and merged optical sections are shown in the right panels. Colocalization analysis of HDAC1 fluorescence signal and the DAPI stain signal was performed with JACoP (ImageJ). Pearson’s Coefficient is presented as the mean of at least two independent experiments ± standard deviation. Optical sections shown are representatives of at least two independent experiments.
(TIF)
