# Supplementary figures and images for: Scaffold dependent histone deacetylase (HDAC) inhibitor induced re-equilibration of the subcellular localization and post-translational modification state of class I HDACs
Source: PLoS One. 2017 Oct 18;12(10):e0186620. doi: 10.1371/journal.pone.0186620 (PMC5646865; doi:10.1371/journal.pone.0186620)

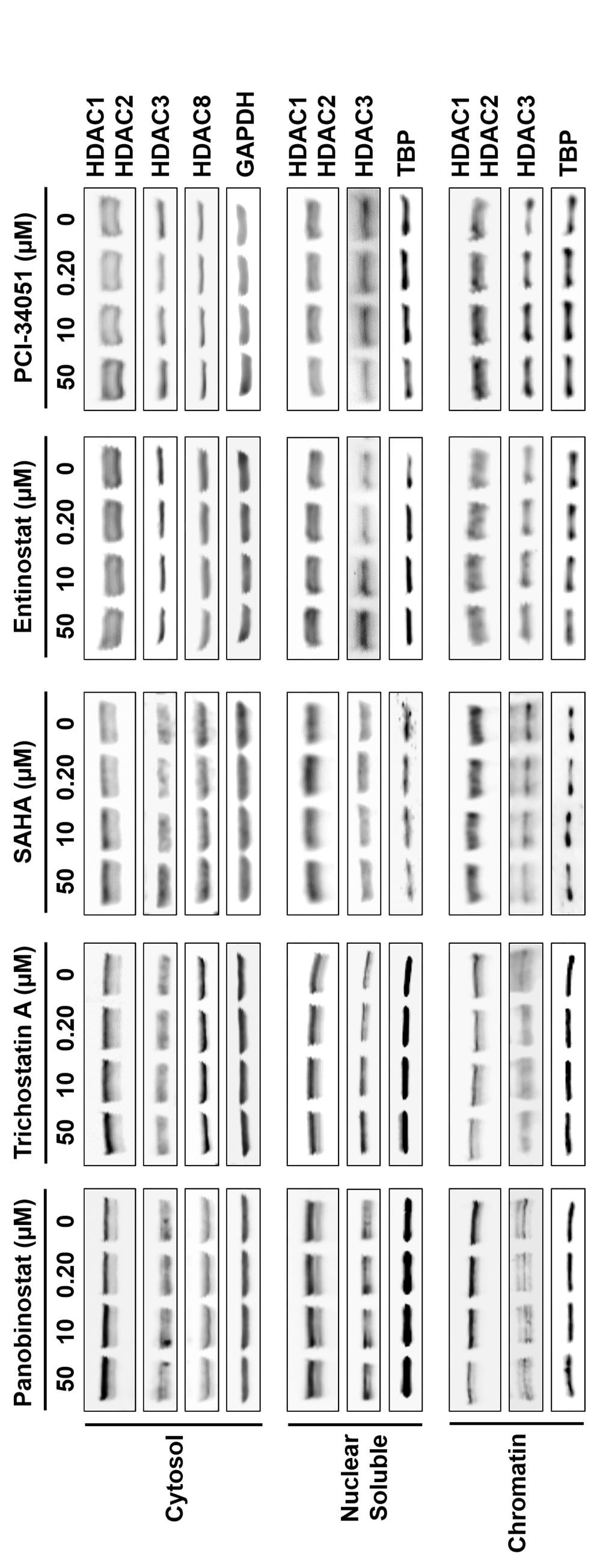

Supplement: S1 Fig — MCF-7 cells were treated with indicated concentrations of panobinostat, trichostatin A, SAHA, Entinostat, or PCI-34051 for 12 hours and then fractionated biochemically. The abundance of class I HDACs was characterized by Western blot analysis in the cytosolic (top panel), nuclear soluble (middle panel), and chromatin bound (bottom panel) fractions. Western blots shown are representative of at least two independent experiments. (TIF) [file pone.0186620.s001.tif]

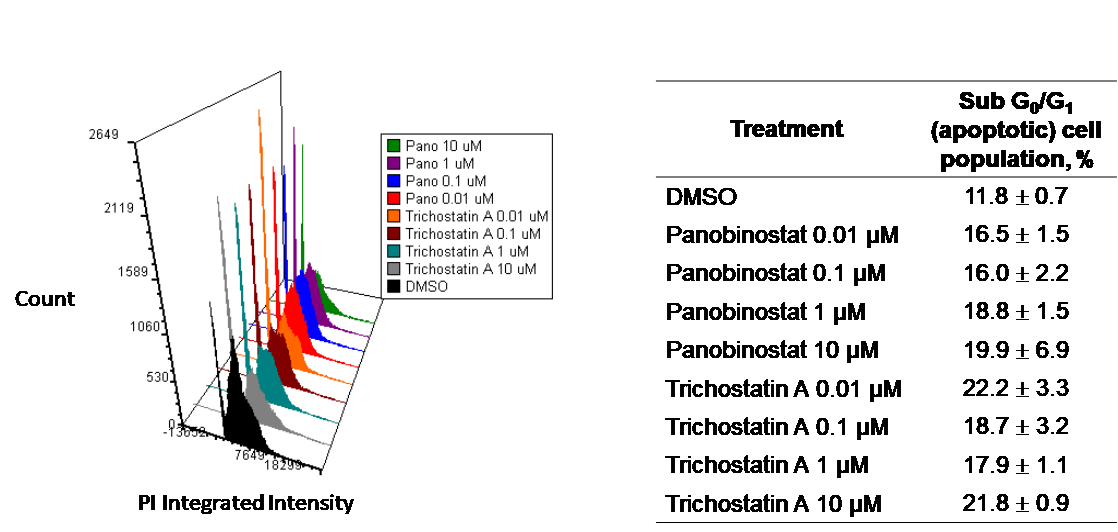

Supplement: S2 Fig — MCF-7 cells were serum starved for 12 hours and treated with indicated concentrations of panobinostat or trichostatin A. Cells were fixed, stained with propidium iodide (PI), and cell cycle analysis was conducted with Celigo image cytometer. Three-dimensional plot on left shows integrated PI intensity and table on right shows percentage of apoptotic cell population for each treatment. Percentage values are expressed as mean ± standard deviation of three replicates. (TIF) [file pone.0186620.s002.tif]

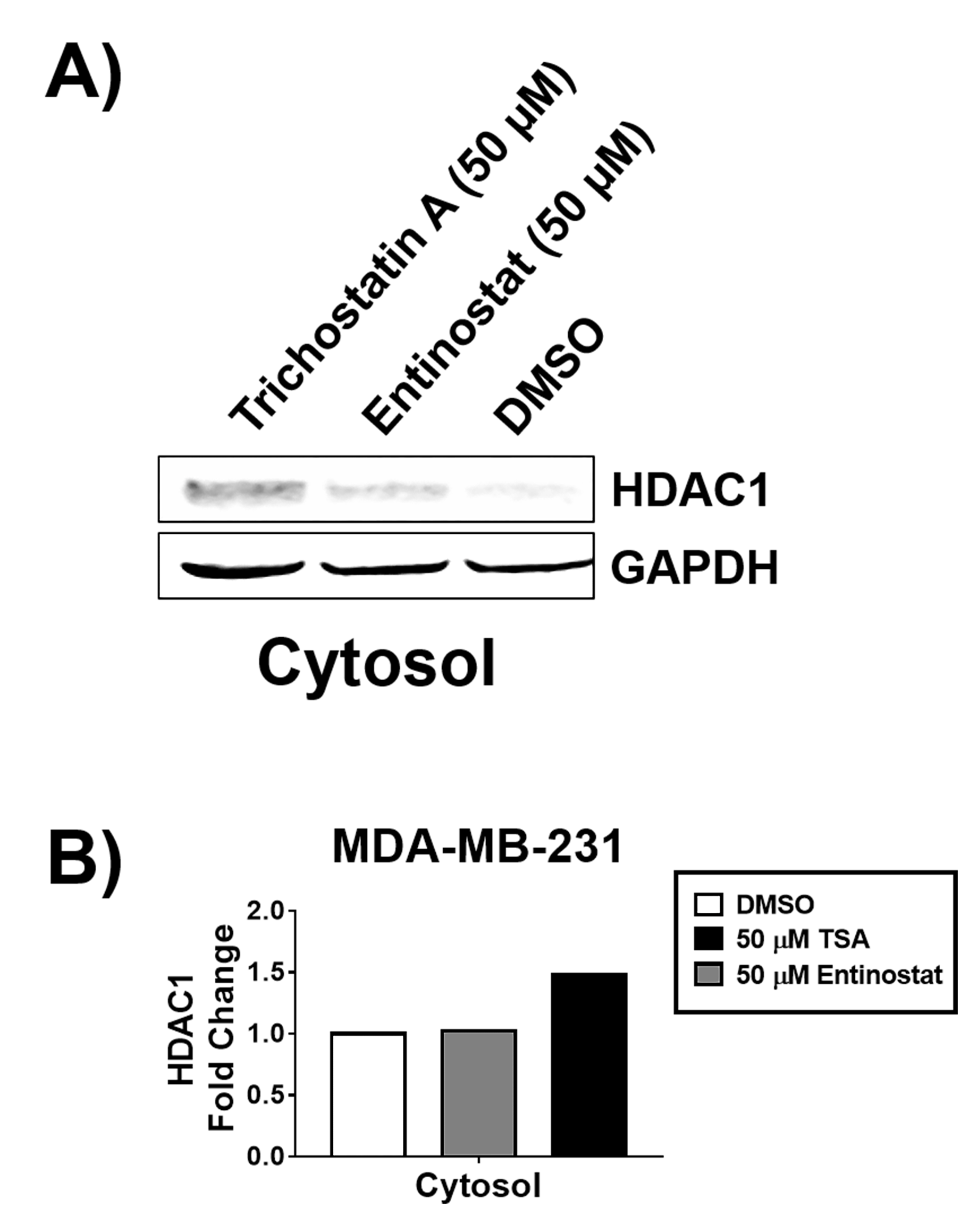

Supplement: S3 Fig — MDA-MB-231 cells were treated with 50 μM trichostatin A or entinostat for 12 hours and then fractionated biochemically. A) The abundance of HDAC1 was characterized by Western blot analysis in the cytosolic (top panel), and chromatin bound (bottom panel) fractions. B) Densitometry analysis of the abundance of HDAC1 normalized to GAPDH loading control. (TIF) [file pone.0186620.s003.tif]

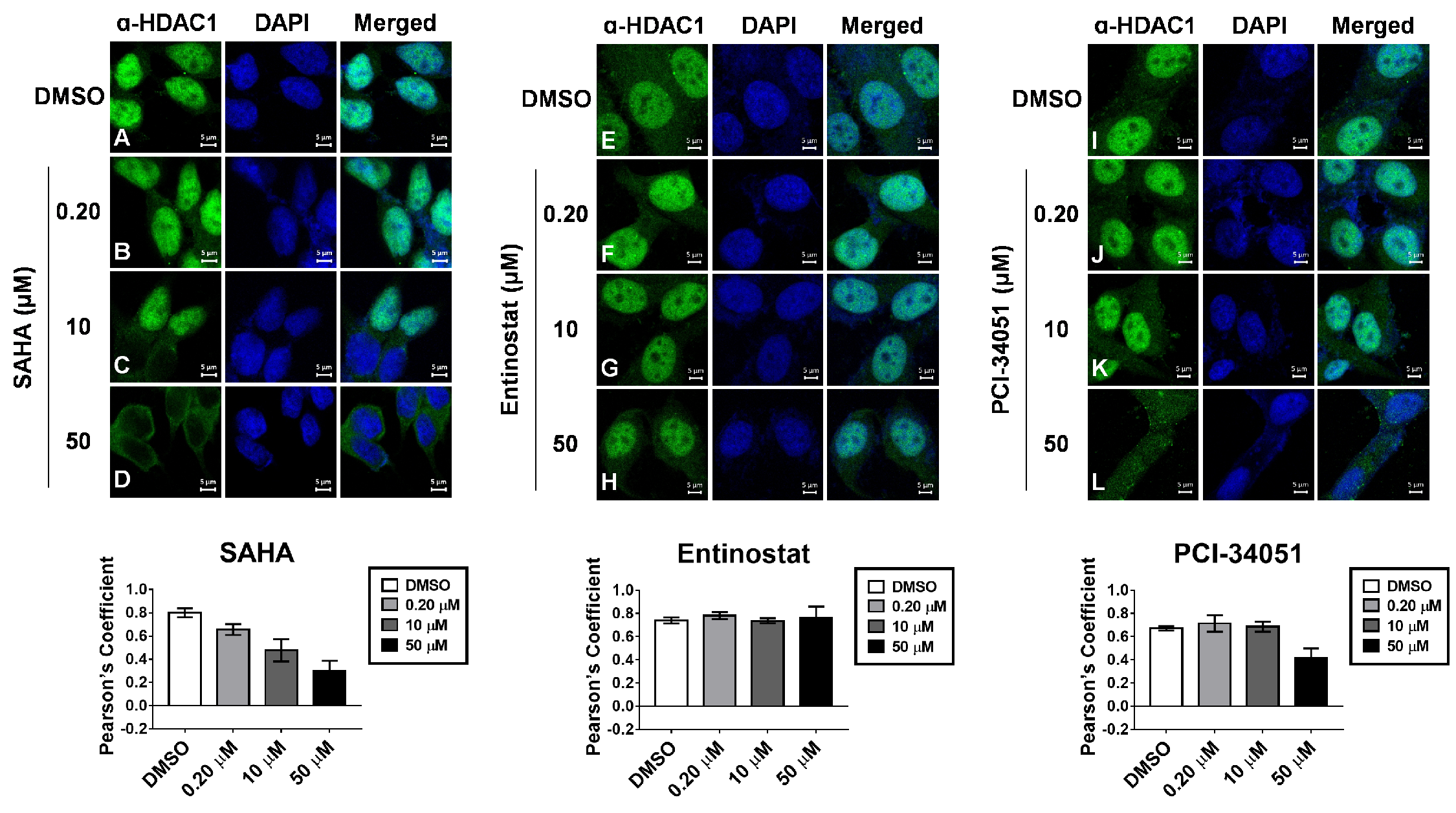

Supplement: S4 Fig — MCF-7 cells were treated indicated concentrations of SAHA (optical sections A-D, respectively), entinostat (optical sections E-H, respectively), or PCI-34051 (optical sections I-L, respectively) for 12 hours, fixed, permeabilized and optical sections were obtained by laser scanning confocal microscopy. Fluorescence signal for HDAC1 is shown in green (left panels), DAPI staining is shown in blue (middle panels), and merged optical sections are shown in the right panels. Colocalization analysis of HDAC1 fluorescence signal and the DAPI stain signal was performed with JACoP (ImageJ). Pearson’s Coefficient is presented as the mean of at least two independent experiments ± standard deviation. Optical sections shown are representatives of at least two independent experiments. (TIF) [file pone.0186620.s004.tif]
